# The Fountain of Age: A Remarkable 3D Shape that Portrays Health and Functional Differences among the European Elderly

**DOI:** 10.3390/ijerph110404078

**Published:** 2014-04-14

**Authors:** Stef van Buuren, Rom Perenboom

**Affiliations:** 1 Department of Life Style, Netherlands Organisation for Applied Scientific Research TNO, P.O. Box 2215, 2301 CE Leiden, The Netherlands; E-Mail: rom.perenboom@tno.nl; 2 Faculty of Social and Behavioural Sciences, University of Utrecht, P.O. Box 80140, 3508 TC Utrecht, The Netherlands

**Keywords:** elderly, health, homogeneity analysis, SHARE, GOAL profiles, mobility, monitoring, personalized medicine, data reduction

## Abstract

There are very few norms to evaluate and monitor the health and functioning of the elderly. This paper proposes a compact spatial representation of 25 health measurements of European citizens older than 50 years. Data from 44,285 unique individuals were obtained from the EU-wide *Survey of Health*, *Ageing and Retirement in Europe*(SHARE) data collected in 2004–2007 and were analyzed by homogeneity analysis, a form of non-linear principal components analysis. The resulting configuration of persons shows a remarkable three-dimensional shape that resembles a fountain. The three components explain 13.7, 5.8 and 4.6 percent of the total variation, respectively. Component 1 is driven by age and by the disabilities that come with old age. Component 2 portrays differences in health that are independent of age, with the high scores in relatively good health, given age. Component 3 distinguishes specific types of functional decline from general complaints that impact on daily life. The shape suggests that the elderly keep on maturing as they grow older, actually becoming more diverse as a group. We show how the solution may be used to develop and support profiles for the elderly. Another potential application is to track the individual development of the elderly, thereby objectifying personalized medicine.

## 1. Introduction

The proportion of elderly people is increasing worldwide and especially in Western societies. In 1900, approximately 13 percent of the European population was older than 50 years. This has risen to 36 percent in 2010. By 2040, about 46 percent of the population will be older than 50 years [1].

The increasing number of elderly persons puts a burden on society at large. Due to this increase and the fact that diseases that used to be fatal are now better treated and managed, the number of older adults with chronic diseases will rise. This increase will lead to a higher demand for care, which will have to be met by a shrinking European work force.

Traditionally, Western society has invested heavily into the treatment of diseases in the aged, but, fortunately, at any age, the clinically treated group represents a relatively small fraction of the total. More importantly, the consequences of aging and diseases show different trajectories, due to the influence of a wide range of mediating factors. One strategy to promote successful aging is therefore to detect negative declines in health and functioning and identify factors that can be positively influenced.

Compared to child growth and development, relatively little is known about the normal aging and developmental processes through the age of 50 years and above. Knowing what constitutes normal development in this group allows us to detect conditions or diseases in individuals before they can become chronic or fatal, resulting in a more informed strategy to promote successful aging.

This paper proposes a compact spatial representation of the health of European citizens older than 50 years, with the objective of providing a simple metric to construct norms for evaluating and monitoring the health of the elderly. Section 2 describes the data used in this paper, how the material was selected and outlines the statistical methodology used. Sections 3.1 and 3.2 form the key parts of this report. Section 3.1 describes a fountain-like 3D shape formed when all individuals are plotted as points. Section 3.2 provides an interpretation of the three axes of the configuration. Section 5 discusses the implications and potential uses of the fountain-like shape.

## 2. Data and Methods

### 2.1. Data Source

Data are used from the *Survey of Health*, *Ageing and Retirement in Europe* (SHARE) (see http://www.share-project.org) [2,3]. SHARE is a multidisciplinary and cross-national panel for collecting micro data on health, socio-economic status and social and family networks on citizens aged 50 years and over living in 19 European countries. Data collection was started in 2004 and has continued since then in five waves, resulting in public access data obtained through approximately 150,000 interviews on 85,000 individuals. This paper uses data collected during Wave 1 (in 2004, *n* = 31,115) and Wave 2 (in 2007, *n* = 34,415), obtained from 44,799 unique individuals.

### 2.2. Participants

To understand the normal development of aging in the elderly, the age of 50 years represents a convenient starting point. At the age of 50, a large part of the population is still in good health, active and employed. For individuals who were interviewed twice, in both Wave 1 and Wave 2, only the data from the first wave were used. Thus, the data consist of a cross-sectional sample of European elderly from the period 2004–2007, resulting in 44,799 individuals. The records for 514 individuals (1.2 percent) who had fewer than 12 (out of 25) observed data values were deleted. Six interviews were deleted because of inconsistent or extreme data patterns. The statistical analysis was based on data from 44,285 unique individuals.

### 2.3. Measurements

The SHARE dataset contains a wide variety of measurements. Two experts on health and its consequences in the aging population independently drafted an initial list of variables from the SHARE database that measured an aspect of health. This combined list contained 63 potentially relevant variables for inclusion. It was circulated among the participants of the European project, *Growing Older*, *staying mobile Transport needs for an aging society* (GOAL) (see http://www.goal-project.eu) [4], and supplemented with additional potentially relevant variables. After two consultation rounds, the list was reduced to 51 variables, which was further reduced to 25 variables by calculating index variables of similar information (e.g., common daily activities, medication, mobility, eyesight, and so on). Table 1 provides an overview of the selected variables, both with their names in SHARE and in GOAL, including seven indices that were calculated from the original material.

### 2.4. Statistical Analysis

The relations between the 25 measures were summarized by a three-dimensional homogeneity analysis [5]. The objective of the analysis was to derive mutually uncorrelated summary variables, known as principal components, that explain as much as possible the variation of the data. No assumptions are made about the ordering of the categories. The analysis quantified each category such that the correlation with the principal component is maximized. Each component obtained its own set of quantifications, so that different components uncover different nonlinear relations. A solution with three components was chosen, because each component could be well interpreted.

Homogeneity analysis results in a biplot, a representation of both the rows (persons) and the columns (variables) of the data in the same low-dimensional space. We refer to the relevant literature for more details about the interpretation of biplots [5,6].

Calculations were done in R3.0.2 [7] using the package, homals [8]. Various graphical displays were used to obtain insight into the most important sources of variation between the elderly. We used the *k*-means algorithm to cluster person points into five groups. The number of clusters was varied between four and seven. The solution consisting of five clusters was elected by subject matter experts as being most useful for the purposes of discriminating profiles in mobility and transportation, as was the intention of the GOAL project.

## 3. Results

By construction, each of the three components is a summary of the 25 measures of Table 1. The interpretation of the three principal components is as follows:

1. Component 1 is driven by age and by the disabilities associated with increasing age;
2. Component 2 portrays differences in health that are independent of age, with the high scores in relatively good health, given age;
3. Component 3 distinguishes specific types of functional decline (cataract, cognitive decline, vision, hearing) from general complaints that impact on daily life (pain, fatigue, sleeping problem and breathlessness).

Each person has a score of each of the three components. Section 3.1 reveals the unique fountain-like shape of the resulting three-dimensional scatterplot of persons. Section 3.2 describes the interpretation of the components in more detail.

**Table 1:** Measures selected from the *Survey of Health*, *Ageing and Retirement in Europe* (SHARE) database. Seven variables are constructed as an index of SHARE variables.

**Number**	**Name**	**Categories**	**Description**
1	age	5	Age at interview
2	sex	2	Gender
3	education	19	Highest educational degree obtained (dn010_)
4	partner	3	Living with spouse/partner (mstat)
5	parkinson	2	Parkinson disease (ph006d12)
6	cataract	2	Cataracts (ph006d13)
7	pain	2	Pain in back, knees, hips or other joint (ph010d1)
8	sleep	2	Sleeping problems (ph010d1)
9	dementia	2	Alzheimer's disease, dementia (ph006d16)
10	fatigue	2	Fatigue (ph010d12)
11	limited	3	Limited activities (ph005_)
12	health	6	General health (ph002_, ph003_)
13	swollenlegs	2	Swollen legs (ph010d5)
14	breathless	2	Breathlessness (ph010d3)
15	hearing	5	Index (ph046_, ph047_r, ph055_, ph056_)
16	eyesight	6	Index (ph042_, ph043_, ph044_)
17	mobility	12	Index (ph048d1, ph048d2, ph048d3, ph048d4, ph048d5, ph048d6, ph048d7, ph048d8, ph048d9, ph049d2, ph049d5)
18	usesaid	2	Index (ph059d1, ph059d2, ph059d3, ph059d4, ph059d5)
19	canusemap	2	Can use a map in an unfamiliar location (ph049d7)
20	falling	4	1 + sum (ph010d7, ph010d8, ph010d9)
21	drugs	5	Index (ph011d7, ph011d8, ph011d9, ph011d10)
22	earning	7	Average of ep205ub and ep207ub (0 = low, 7 = high)
23	cars	4	Number of cars (AS049_)
24	endsmeet	4	Able to make ends meet (co007_)
25	area	5	Area where respondent lives (ho037_)

### 3.1. Fountain of Age

**Figure 1:**
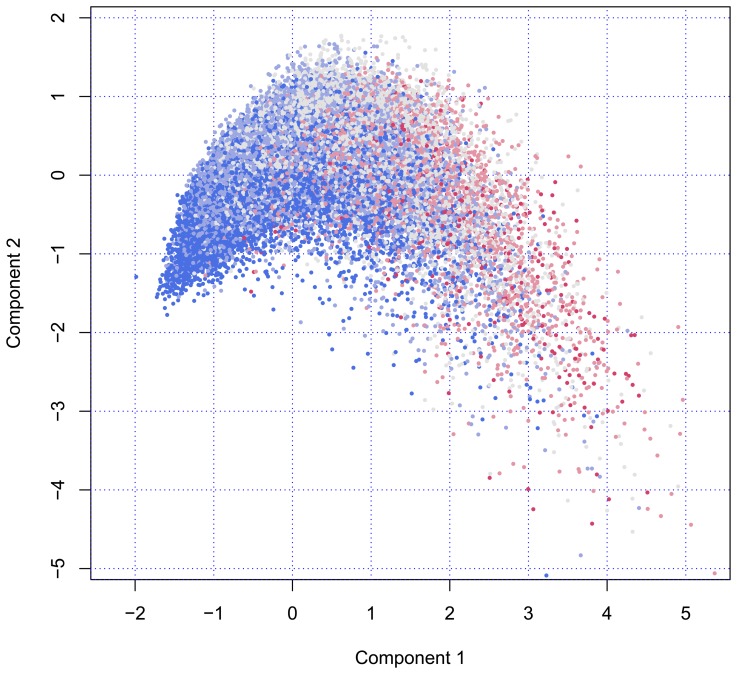
The locations of 44,285 elderly persons on the first two principal components. Points are colored according to age group (blue = 50−59 years, red = 80−105 years) (side view).

Figure 1 plots the scores of all individuals on Component 1 (on the horizontal axis) and Component 2 (on the vertical axis). The configuration of person points in the scatterplot starts at the left at coordinate (*x* = −1.5, *y* = −1.5). There is almost no variation around that point, an unusual feature that is caused by a floor effect. The vertical spread of the data points increases as we move from left to right.

The number of persons in the age categories are equal to 17,355 (50–59), 13,803 (60–69), 9262 (70–79), 3442 (80–89) and 423 (90–105). The color indicates that the age composition changes dramatically; so, near the pivotal point of (*x* = −1.5, *y* = −1.5), most persons are aged 50–59 years, with older persons located more to the right. Figure 1 illustrates that variation among older persons is much larger than among younger persons. Another interesting aspects is that there seems to be some optimum on Component 2. From the left to the right, the cloud rises and reaches a maximum before gradually falling off at the right-hand side.

Figure 1 may be perceived as a fountain, viewed from the side. The “nozzle” located at (*x* = −1.5, *y* = −1.5) produces a high-pressure flow of young, fresh and healthy persons, whereas individuals further away from the nozzle spread out into space, just like real water would do, eventually dissolving into drops.

**Figure 2:**
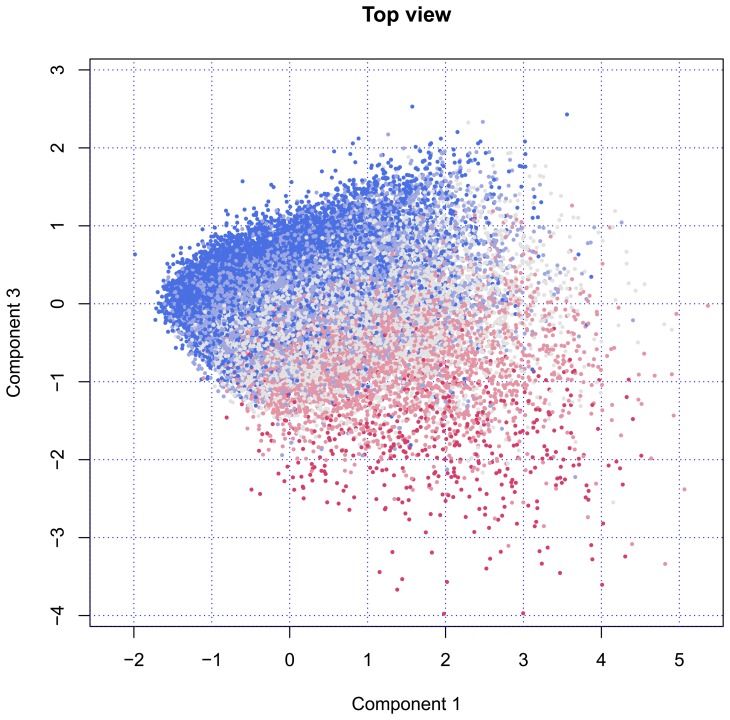
Locations on Components 1 and 3; points are colored according to age group (blue = 50–59 years, red = 80–105 years) (top view).

Figure 2 plots the same data, but with a view from the top. This introduces Component 3, which replaces Component 2, as plotted in Figure 1. When viewed from above, the points rise at the left-hand side and decrease at the right-hand side. Both Components 1 and 3 are related to age, where Component 3 clearly separates younger and older individuals in upper and lower regions. Although the group is much smaller, the variation among the older persons is much larger than among the younger ones.

**Figure 3:**
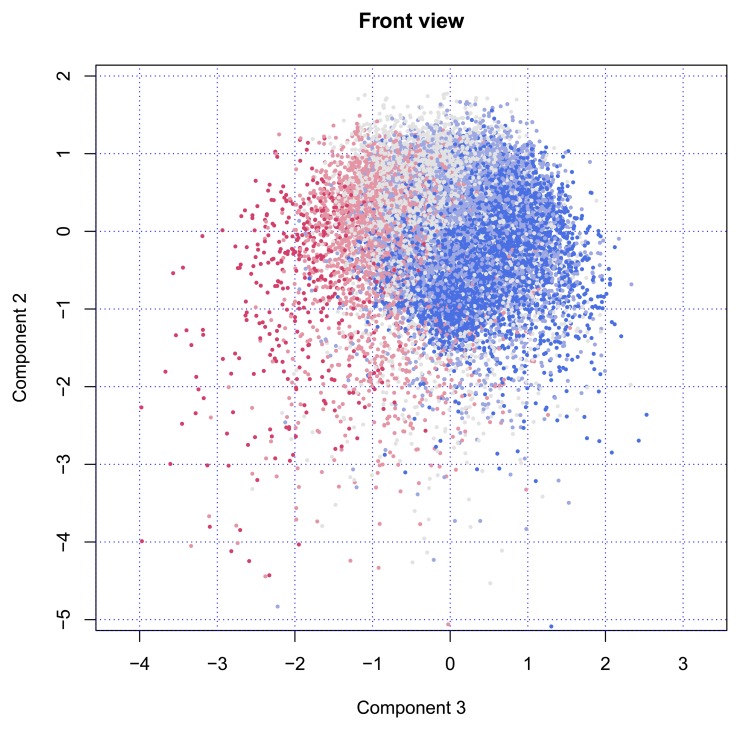
Locations on Components 2 and 3; points are colored according to age group (blue = 50–59 years, red = 80–105 years) (Front view).

Finally, Figure 3 shows the cloud of points from the front side. In this position, the fountain will spray the water in our direction. The nozzle is still visible in the back at location (*x* = −1.5, *y* = −1.5). The figure clearly illustrates that Component 2 has little to do with age, while Components 3 is strongly age-dependent.

The percentage of variance explained by the first three principal components was 13.7, 5.8 and 4.6 percent, respectively. Thus, the three-dimensional solution accounts for about 24 percent of the total variation, where Component 1 is much more important than either Components 2 or 3.

### 3.2. Interpretation

**Figure 4:**
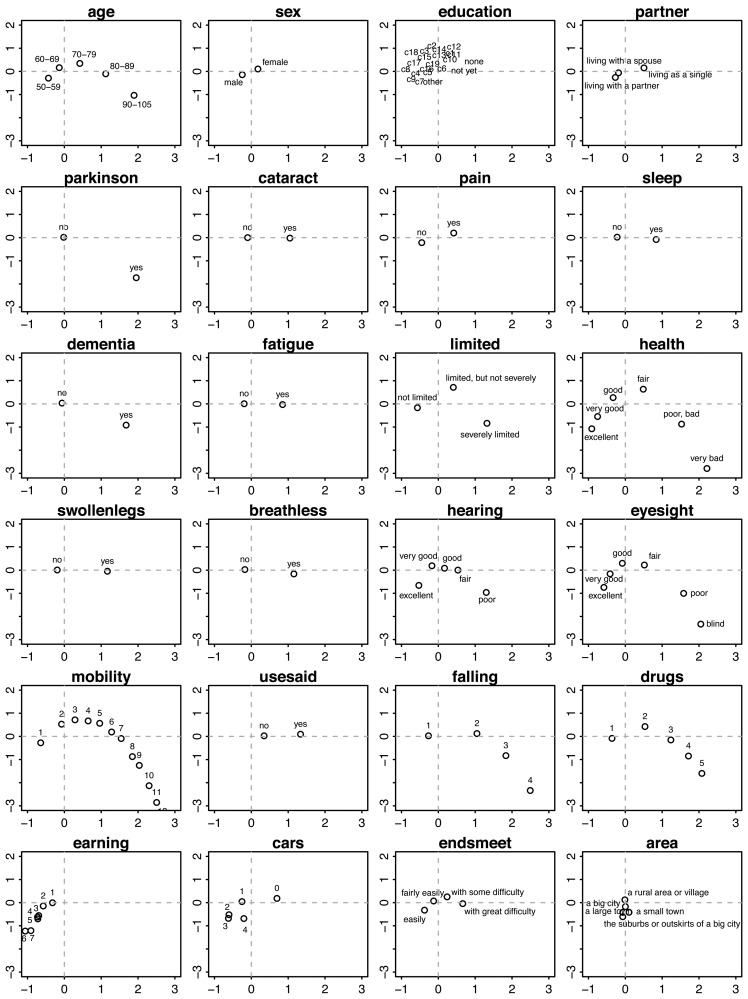
Category quantifications on Component 1 (horizontal) and Component 2 (vertical) per variable (side view).

We used graphs of the category quantifications to interpret each component. Figure 4 displays the category quantifications for Components 1 and 2. Each location corresponds to the average of all persons scoring in that category. For example, if we average the points of all persons aged 50–59 years, then the mean will be located at approximately (*x* = −0.5, *y* = −0.5).

Persons located in the right-lower region in the plane of Component 1 and Component 2 belong to the very old (80+ years), have serious chronic health conditions, like Parkinson disease, cataracts, dementia, deafness and poor eyesight, are severely limited in their activities, experience difficulties with walking, sitting, and so on, have a high risk of falling and use many fall-related drugs.

In contrast, persons on the left-hand side are, on average, younger (50–59 years), live with a partner, are more often male, are not limited in their daily activities, experience no pain, enjoy good to excellent health, have no falling risk, and use no fall-related medication, are highly mobile (in terms of moving around), have higher incomes, possess more cars and live in suburbs and small towns.

People located on the upper side are (on average, aged 70–79 years) more often female, living single, have good to fair health, are bothered by pain and sleeplessness, have swollen legs, are easily out-of-breath, have fair eyesight and use hearing aids, experience few difficulties in getting around, are limited, but not severely, use no drugs that increase the risk of falling, have no car and have some difficulty in making ends meet.

**Figure 5:**
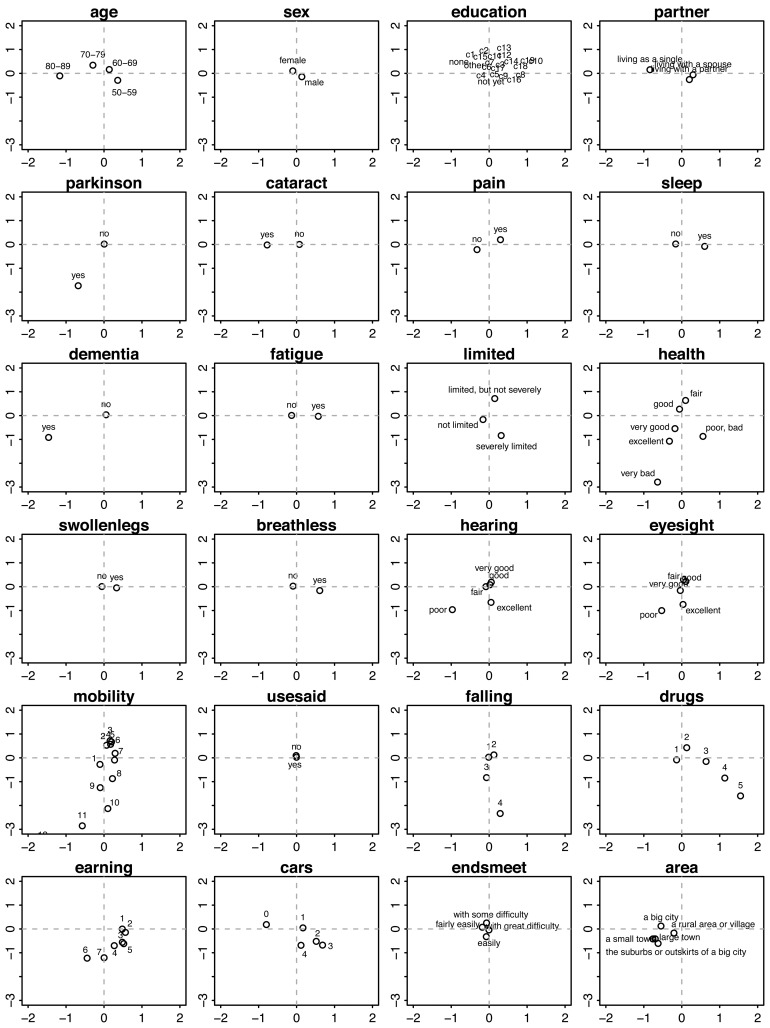
Category quantifications on Component 3 (horizontal) and Component 2 (vertical) per variable (Front view).

In the front view, *i.e.*, in the plane of Component 1 and Component 3 (Figure 5), we find that persons having high values on Component 3 (to the right) are generally younger and live with a partner, are bothered by pain and sleeplessness, have swollen legs, are easily out-of-breath, use many fall-related drugs, have relatively low incomes and possess a car. In contrast, persons with low scores on Component 3 (to the left) are generally older and live along, are at risk for dementia, have cataracts, poor eyesight, poor hearing and somewhat higher incomes.

## 4. Application: Health Profiles

Although the spectrum of variation between persons is clearly continuous, interpretation and communication can be easier by distinguishing a small number of health and functioning profiles described by verbal labels. People within the same profile are considered more similar than people belonging to different profiles.

**Figure 6:**
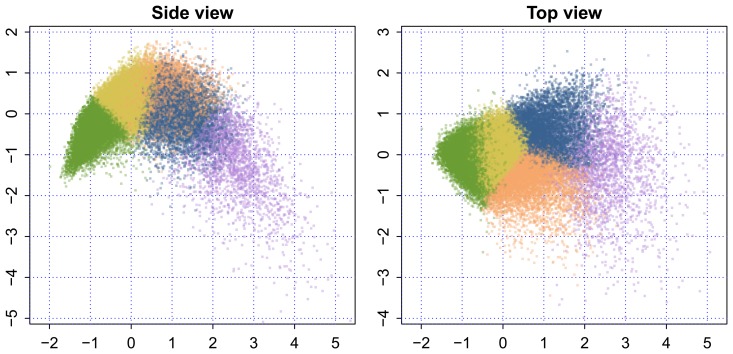
Side and top view of persons colored by allocated profile.

Figure 6 is a representation of the 3D shape, where each point is colored by one of five colors. The color groups were formed by the *k*-means clustering algorithm. The five clusters are a readily interpretable summary of the profiles of the European elderly. The following group labels were adopted by the GOAL project: [9]

1. Green: *Fit as a fiddle*. People in this group do not consider themselves as old, live with family or a partner, are still employed, have a good social network and enjoy high life satisfaction and autonomy. Transition points include retirement, severe illness and the birth of grandchildren (*n* = 16,371).
2. Yellow: *Happily connected*. People in this group live in a partnership, have a very good social network (family, care for grandchildren, friends, clubs, volunteering), a high quality of life and autonomy, are mostly retired and emphasize staying fit and healthy as important goals. Transition points include severe illness or injury and the loss of social contacts (family member or friends) (*n* = 14,067).
3. Orange: *An oldie*, *but a goodie*. People in this group are living alone more often, are more often female, are retired, may experience some financial problems and enjoy satisfaction, self-efficacy and autonomy. Transition points include severe illness and the death of a close person (*n* = 5,910).
4. Blue: *Hole in the heart*. People in this group have a lower quality of life, are mostly retired, are more often sick, have fewer social contacts and activities and run a risk of loneliness. Transition points include illness, death of a close person (partner), new social contacts or the loss of social contacts (*n* = 5,689).
5. Purple: *Care-full*. People in this group often live without partner in a family member's home or a home for the elderly, live more often in assisted living areas, have low autonomy and life satisfaction, need (nursing) care, run the risk of social exclusion and primarily are concerned with passive activities within their homes, like receiving visitors. Transition points include severe illness, dependency on others and the loss of social contacts (*n* = 2,249).

The precise relation between the SHARE data sample and the target population of European elderly persons is very complicated and, in fact, differs between countries [3]. The unweighted counts as presented above therefore represent only an approximation of the true relative sizes of the five groups in the European population.

## 5. Conclusions

Our model represents the health and functional status of European citizens aged 50 years and older by three components. The collection of individual data values on these components forms a remarkable fountain-like 3D shape. The younger, active and healthy people are located close to the “nozzle”, while the older and disabled spread out as they age. The increased scatter suggests that the elderly keep on maturing as they grow older and, as a result, actually become more diverse as a group.

The parabolic shape on Component 2 suggests that there might be some sort of optimum. It is not yet clear what the nature of such an optimum could be and whether it would be beneficial in trying to attain high values on Component 2. This is an area for further research.

The representation as presented here provides an enormous reduction of the 25 health and functioning variables listed in Table 1 to just three continuous measures, each of which has a clear interpretation. Perhaps an amount of 24 percent explained variance does not sound impressive, but keep in mind that the 25 input variables are quite diverse. Its virtue is that it enables us to form a mental image of the major differences in the health and functional status in the elderly.

The metric can be helpful for various purposes. First of all, it is straightforward to cluster persons in a small number of health profiles. Such health and functioning profiles can be used for intervention development and for policy setting and evaluation.

Another potential application is tracking the individual development of the elderly. In contrast to child development, good references of normal maturation and aging processes in the elderly are not available. We could systematically monitor the maturation of the elderly on a periodical basis, preferably combined with a rational approach per phase [10]. Individual maturation can be visualized by plotting the measured health status at different time points and connecting the dots. The resulting track may be used to predict the likely future course of that individual on the three components, in comparison with other individuals.

An even more advanced use would be to simulate the effects of a potential intervention (e.g., a surgery or a reduction in blood pressure) on the future course, for example as calculated through curve matching [11]. Such prospective evaluations may provide an objective handle for personalized medicine [12]. Creative researchers will undoubtedly be able to find other applications.

An appealing feature of our solution is that it is based on a large, continuing and open European data source. The shape we found is thus easily reproducible and extendible by others. We believe that the fountain shape is universal and not particularly tied to one country, one cohort or one particular set of measures, but we have not yet actually tested this. For this analysis, the included variables are expected to have an association with the mobility of the elderly in its broadest sense: from walking and cycling, to car driving and making use of means of public transportation. Therefore, not all items on health and functioning in the SHARE database are included. The wide availability of the SHARE data makes it easy to explore alternative choices.

This is the first time that we have seen this fountain-like shape. We believe that the shape is more than just an oddity, and we hope that it may inspire novel ways of thinking about the health of elderly people.
